# Insolvenzrelevante Fußball-Verbandsregularien in Deutschland: Ökonomische Besonderheiten, Fehlanreize und Reformansätze

**DOI:** 10.1007/s12662-020-00666-7

**Published:** 2020-08-04

**Authors:** Daniel Weimar

**Affiliations:** Akademischer Rat, Mercator School of Management, General Business Administration, University of Duisburg-Essen, Lotharstraße 65, 47057 Duisburg, Deutschland

**Keywords:** Insolvenz, Sportfinanzierung, Insolvenzordnung, Insolvenzsanktionen, Fußballsystem, Insolvency, Sports finance, Insolvency policy, Insolvency penalties, Football system

## Abstract

Aufgrund zahlreicher ökonomischer Besonderheiten des Fußballmarktes ist der Umgang mit insolventen Fußballclubs durchaus komplex. Als eine Reaktion auf diese Besonderheiten agieren Fußballverbände mit „Sonder-Insolvenzklauseln“, um den sportlichen Wettbewerb zu sichern. Studien attestieren diesen Verbandsregularien jedoch einen teilweisen Widerspruch zur Insolvenzordnung. Auch von Seiten der Fußballclubs wächst die Kritik gegen die Sanktionspraxis. Ökonomisch-rechtliche Auseinandersetzungen mit derartigen Verbandsregularien sind jedoch rar. Daher diskutiert der Beitrag Besonderheiten, Fehlanreize und potenzielle Reformansätze von Verbandssanktionen im Zusammenhang mit Insolvenzverfahren von Fußballunternehmen in Deutschland. Im Ergebnis werden Nachteile eines festen Punktabzuges herausgestellt und Alternativen diskutiert. Ferner wird herausgearbeitet, dass die Übertragung von Ligarechten an Nachfolgeclubs unterbunden und vorinsolvenzliche Verfahren berücksichtigt werden sollten. Da in der 1. Bundesliga keine und in der 2. Bundesliga nur zwei Insolvenzverfahren seit 1995 durchgeführt wurden, entfalten die Ergebnisse besondere Relevanz für die professionellen und semiprofessionellen Ligen drei bis fünf.

## Abstract (en)

Due to numerous economic peculiarities of the football (soccer) market, dealing with insolvent football clubs is complex. To ensure the call for fair competition, German football associations implemented special insolvency policies. However, past research has discussed partial contradiction of these association regulations to the official insolvency law. The criticism of competitor clubs against the sanctioning practice is also increasing. Due to the lack of economic and legal scientific discussion, this article discusses economic peculiarities, disincentives and potential reform approaches of association norms associated with insolvency proceedings of football clubs in Germany. The disadvantages of a fixed point penalty are highlighted and alternatives are discussed. Furthermore, the following are also discussed: the transfer of league rights to successor clubs should be prevented and the preinsolvency proceedings should be taken into account. Since there have been no insolvency proceedings in the first Bundesliga and only two in the second Bundesliga since 1995, the results are particularly relevant for the professional and semiprofessional leagues (third to fifth Liga).

Schon der Blick auf statistische Vergleichswerte verdeutlicht: Insolvenzverfahren von Fußballclubs sind durchaus atypisch. Während die allgemeine Insolvenzwahrscheinlichkeit von Wirtschaftsunternehmen in Deutschland 2018 durchschnittlich 0,59 % betrug (DESTATIS, 2019), so lag dieser Wert – bezogen auf deutsche Fußballunternehmen und -vereine (im Folgenden Fußballclubs) der ersten vier Ligen – bei 2,7 % und damit viermal höher (2017 sogar 3,4 %; Szymanski & Weimar, 2019, dabei jedoch keine Insolvenz in der 1. und 2. Bundesliga).^1^ Kommt es zur Eröffnung eines Insolvenzverfahrens, bildet das Insolvenzplanverfahren als Sanierungsinstrument der Insolvenzordnung (InsO) die seltene Ausnahme außerhalb des Sports (ca. 2 %, IT.NRW, 2017). Im Gegensatz dazu liegt die Quote der Insolvenzplanverfahren bei Fußballclubs etwa 17-mal höher (47,9 % von 1999 bis 2018, Szymanski & Weimar, 2019).^2^ Eine gesonderte ökonomisch-rechtliche Auseinandersetzung mit Insolvenzverfahren von Fußballclubs scheint vor diesem Hintergrund durchaus interessant.

Obwohl im Fall eines Insolvenzverfahrens eines Fußballclubs zunächst die InsO gilt, so sind im deutschen Fußballsystem zusätzlich gesonderte Verbandsstatuten zu beachten, welche sportliche Sanktionen vorsehen. Diese sehen entweder einen 9‑Punkte-Abzug oder einen Zwangsabstieg vor. Die Vereinbarkeit solcher Sanktionsklauseln mit geltendem (Insolvenz‑)Recht kann durchaus angezweifelt werden, wurde doch mit Inkrafttreten der InsO 1999 die rechtliche Grundlage zum Erhalt eines insolventen Schuldners und zu dessen Fortführung geschaffen. Da die Verbandsregularien jedoch zusätzliche sportliche Sanktionen vorsehen, welche genau diese Fortführung gefährden („gravierendes Sanierungshindernis“, Siemon, 2015), zeichnet sich ein Widerspruch ab, welcher sowohl von rechtswissenschaftlichen Untersuchungen (Hörnig & Knauth, 2018; Knauth, 2018) als auch von gerichtlichen Ausführungen (LG Berlin 26.02.2015 – 35 O 56/15) gestützt wird.

Trotz der durchaus reichen^3^ Historie an wirtschaftswissenschaftlichen Debatten hinsichtlich der finanziellen Besonderheiten von Fußballclubs, sind Insolvenzen von Fußballclubs weiterhin ein von der ökonomischen Forschung relativ unangetasteter Forschungsbereich. Rechtsanalytische Auseinandersetzungen liegen erst ab 2013 vor (Korff, 2013; Lambrecht & Reiter, 2013; Fröhlich & Fröhlich, 2013), wobei erst Knauth (2018) sowie Hörnig und Knauth (2018) die relevanten Verbandsparagraphen kritisch diskutieren. Wirtschaftswissenschaftlich analysierten zunächst Beach, Horsman, und Magraw (2010) sowie Szymanski (2017) Insolvenzvorfälle in England, bevor Scelles, Szymanski, und Dermit-Richard (2018) Insolvenzverfahren in Frankreich untersuchten. Für den deutschen Fußballmarkt haben Weimar und Szymanski (2019) erstmals empirische Analysen vorgelegt. Eine kritisch-ökonomische Analyse der einschlägigen Verbandsregularien sowie eine Erarbeitung von Reformvorschlägen – unter Berücksichtigung der Besonderheiten des Fußballmarktes – liegen bisher nicht vor und sollen daher nachstehend skizziert werden.

Eine derartige Auseinandersetzung erscheint aus vier Aspekten heraus interessant. Erstens verstärkt sich auf Seite der (Mit‑)Wettbewerber (Ligakonkurrenten) zunehmend die Kritik hinsichtlich eines festen 9‑Punkte-Abzuges für insolvente Clubs. Zweitens gelten aktuell noch keine einheitlichen Insolvenzsanktionen innerhalb des deutschen Fußballsystems, weshalb zeitnah Vereinheitlichungstendenzen einsetzen könnten und somit Ideen zur Neugestaltung nötig werden. Drittens scheinen die finanziellen Schwierigkeiten von deutschen Fußballvereinen nicht abzunehmen. Es besteht daher die Frage, warum die Verbandssanktionen – als auch die Insolvenzordnung – keine ausreichende Abschreckungskraft entfalten bzw. wie an dieser Stelle effektiver aus Verbandssicht entgegengesteuert werden kann. Viertes ist durch die COVID-19-Pandemie 2020 deutlich geworden, dass eine komplette Außerkraftsetzung von Insolvenzsanktionen von Teilen der Klubs kritisch gesehen wird. Damit ergibt sich aus diesem Aspekt heraus die Frage, weshalb eine derartige Forderung nach Strafen von Seiten der Klubs besteht und wie diese besser ausgestaltet werden könnten.

## Besonderheiten des deutschen Fußballmarktes

Zentral für die nachstehende Diskussion eines sachgerechten Umgangs mit insolventen Fußballclubs sind einige charakteristische Besonderheiten des Marktbereiches Fußball. Neben staatlichem Recht sind Fußballclubs zunächst an autonome Ordnungen der den Wettbewerb austragenden Verbände gebunden (z. B. Spielordnung, Rechts- und Verfahrensordnung). Dabei gelten in der 1. und 2. Bundesliga die Ordnungen der Deutschen Fußballliga (DFL), in der 3. Liga die Statuten des Deutschen Fußballbundes (DFB), auf Regionalebene die Ordnungen der Regionalverbände und auf den nachgeordneten Landesebenen die Regulierungswerke der jeweiligen Landesverbände.

Ferner gilt für Fußballmärkte – im Gegensatz zum Monopolbestreben auf Nicht-Sportmärkten – generell das Prinzip der assoziativen Konkurrenz (Schellhaaß & Enderle, 1998). Demnach ist ein Wettbewerb nur dann für alle teilnehmenden Clubs nutzenmaximierend, wenn ein ausgeglichenes Konkurrenzniveau vorherrscht (El-Hodri & Quirk, 1971). Dies gilt insbesondere aus Sicht der wettbewerbsorganisierenden Verbände, welche zur Einnahmen- und Reichweitenmaximierung (Zuschauer, TV-Medien) einen spannenden, fairen und ausgeglichenen Wettbewerb anstreben (Gaum, 2017; Schreyer, Schmidt, & Torgler, 2018). Aus diesem Antrieb heraus ist auch ein Verbandsanspruch an gleiche Grundvoraussetzungen für alle teilnehmenden Clubs abzuleiten (Preuss, Haugen & Schubert, 2014).

Drittens sind Auf- und Abstiege (als positive und negative Schocks) für Fußballclubs seit jeher ein besonderes Marktcharakteristikum (Noll, 2002). Besonders Abstiege führen zu plötzlichen Reduzierungen von Einzahlungen, wobei sich aufgrund relativ fixer Auszahlungsverpflichtungen (z. B. Gehälter von Spielern, Verwaltung und Trainern, Spielbetrieb, Zinsen für Darlehen) finanzielle Spannungen verstärken und somit die finanzielle Unsicherheit steigt (Szymanski & Weimar, 2019).

Basierend auf der Tatsache, dass die *Produktion* eines Fußballspiels primär durch Humanressourcen realisiert wird, entlohnen Clubs marginale Qualitätsunterschiede von Spielern tendenziell mit überproportionalen Gehaltssprüngen (Frick & Klaeren, 1997; Franck & Nüesch, 2012; Stigler & Becker, 1977). In der Folge investieren alle Fußballclubs simultan in die Maximierung des eigenen sportlichen Erfolgs (Gehälter, Transferzahlungen oder eigene Ausbildung in einem Nachwuchsleitungszentrum, Wallebohr & Daumann, 2019), wodurch regelmäßig über die marginale Grenzproduktivität einzelner Spieler hinaus investiert und somit ein ökonomisches *Rattenrennen* initiiert wird (Akerlof, 1976; Franck, 2010). Dies induziert bei Abweichung von Saisonzielen – und systematisch verfehlt immer eine vorgegebene Anzahl an Clubs das Saisonziel Klassenerhalt/Aufstieg – regelmäßig Liquiditätslücken (Szymanski, 2015; Szymanski & Weimar, 2019). Ein besonderer bilanzieller Umstand in diesem Zusammenhang ist die eingeschränkte Aktivierung von vertraglichen Verfügungsrechten an Spielern (sog. Spielerrechte als immaterielles Vermögen). Grundsätzlich können Spielerrechte nur mit den Anschaffungsaufwendungen (z. B. Ablösesumme, Beraterhonorar, Ausbildungsentschädigungen) bilanziert werden (BFH-Urteil vom 14.12.2011, I R 108/10, lineare Abschreibung über Vertragslaufzeit). Spielerrechte an Jugendspielern oder ablösefrei erworbenen Spielern bleiben stille Reserven und können nur *aktiviert* werden, sofern bei einem Wechsel (oder einer Vertragsverlängerung) ein laufender Vertrag besteht und Anschaffungsaufwendungen anfallen (ohne laufenden Vertrag wechseln Spieler ablösefrei; BFH-Urteil vom 14.12.2011, I R 108/10).

Fünftens sind Fans als Konsumenten fußballbezogener *Dienstleistungen* stets durch Emotionen und *Identität* mit einem Club verbunden (Beaton, Funk, Ridinger, & Jordan, 2011; Heinemann, 2001). In diesem Zusammenhang hervorzuheben ist die limitierte Substitutionsmöglichkeit einer Fan-Leidenschaft (Giulianotti, 2005). Dieser Umstand bringt Fans in einen „Lock-in“-Zustand, in welchem sie den maximalen Fan-Nutzen nur mit ihrem bisherigen Club erfahren können. Im Ergebnis lässt sich ein Club bei Misserfolg bzw. Insolvenz nicht ohne substanzielle Nutzeneinbußen *eintauschen* (Koenigstorfer, Groeppel-Klein & Schmitt, 2010). Aufbauend auf dieser Verbundenheit der Fans mit dem Club sind auch andere Stakeholder von einer allgemeinen Fan-Enttäuschung betroffen, denn Fans sind ebenso Wähler, Kunden und Meinungsträger. In diesem Zusammenhang wird daher in bestehenden Studien oft mit dem „Soft-Budget-Constraint“ (Kornai, 1979, 1986) argumentiert und eine gewisse lokale Systemrelevanz von Fußballclubs angenommen, welche die Überlebenswahrscheinlichkeit im Insolvenzfall positiv beeinflusst (Storm & Nielsen, 2012; Szymanski & Weimar, 2019).

Als letzte Besonderheit ist der Umstand anzuführen, dass Insolvenzen im Fußball weniger auf dem totalen Versinken der allgemeinen *Nachfrage* nach Fußball beruhen, sondern vorwiegend eine (kurzfristige) liquiditätsbezogene Ursache (oder im Fall der COVID-19-Pandemie exogene Ursachen) haben. Dies beruht auf der Tatsache, dass die Ligen als Wettbewerbsmärkte – und somit auch die ligabezogene Nachfrage – unabhängig von der Insolvenz eines einzelnen Clubs weiter existieren. Die Nachfragen nach dem clubeigenen Produkt „Fußballspiel“ sind darüber hinaus insofern sicher, als dass gegnerische Teams verpflichtet sind anzutreten und somit auch die gegnerischen Konsumenten (Fans) das Produkt beider Teams konsumieren müssen.^4^ Hinzu kommt, dass der Zugang zu einem größeren Markt (höhere Liga) aufgrund von immer gegebenen Aufstiegsrechten (Markteintrittsbarriere) nie gänzlich ausgeschlossenen ist. Damit ist die Nachfrage nach dem Produkt Fußball zwar geringer in einer tieferen Liga, jedoch wächst die Nachfrage bei einem Aufstieg auch wieder in ähnlichem Verhältnis. Die grundlegende Nachfrage nach Fußball (Teilnahme an einem Fußball-Ligawettbewerb) ist somit stets latent gegeben, wohingegen auf Nicht-Sportmärkten eine einmal versunkene Nachfrage nach einem Produkt nur sehr selten wieder neu wachsen kann (z. B. CD-Player, Polaroid, Bubble-Tea) oder enttäuschte Kunden zu einem Wettbewerber abwandern (im Fußball „Lock-in“).^5^ Zusammen mit dem Umstand, dass im Falle einer Liquidierung von den restlichen Vermögenswerten wie Lizenzen, Verträgen und Spielerrechten nur marginale Veräußerungswerte übrig bleiben würden, ist ein Sanierungsverfahren zur bestmöglichen Gläubigerbefriedigung oftmals einer Liquidierung vorzuziehen (Lambrecht & Reiter, 2013).^6^

## Rechtspraxis zum Ablauf und Sanktionierung einer Insolvenz von Fußballclubs

Fußballclubs sind hinsichtlich der Konsequenzen und des Ablaufes eines Insolvenzverfahrens sowohl an die Insolvenzordnung als auch an die Spielordnung des entsprechenden Verbandes gebunden.

Zunächst regelt die Insolvenzordnung den gesetzlichen Ablauf einer Insolvenz. Entsprechend der Insolvenzordnung müssen Fußballclubs (sowohl Kapitalgesellschaften als auch eingetragene Vereine) mit einer Frist von drei Wochen (§ 15a InsO) bei Illiquidität (§ 17 InsO), bei drohender Zahlungsunfähigkeit (§ 18 InsO) oder im Fall von Überschuldung (§ 19 InsO) einen Antrag auf Eröffnung eines Insolvenzverfahrens beim lokalen Amtsgericht stellen (§ 13 InsO). Antragsberechtigt sind dabei sowohl Gläubiger (im Fußball i. d. R. Sozialkasse, Finanzamt oder Krankenkassen) als auch der Club selber (§ 13 InsO). Der Antrag auf Eröffnung eines Insolvenzverfahrens kann bis zum Zeitpunkt der Eröffnung oder Ablehnung des Insolvenzverfahrens zurückgenommen werden (§ 13 InsO).^7^ Sofern die verbleibende Insolvenzmasse nicht die Planprozesskosten decken wird, kommt es zur Liquidierung des Clubs (Abweisung mangels Masse) aus dem amtlichen Vereins‑/Firmenregister und auch aus dem DFB Clubregister (§ 26 InsO). Ist ausreichend Insolvenzmasse vorhanden, wird das Verfahren eröffnet (§§ 27–30 InsO) und ein Insolvenzverwalter ernannt (§ 56 InsO). Seit 2012 kann in bestimmten Fällen der alte Vorstand das Verfahren in Eigenverwaltung unter Aufsicht eines Sachverwalters führen (§ 270 InsO). Anschließend werden alle Gläubiger zu einer Versammlung gerufen (§ 74 InsO), um über einen durch den Club und den Insolvenzverwalter ausgearbeiteten Sanierungsplan abzustimmen (§ 218 InsO). Neben der konkreten Sanierung regelt der Insolvenzplan auch die Insolvenzquote bzw. den Auszahlungsbetrag aus der Insolvenzmasse (meistens deutlich unter 5 %; auch ein Null-Plan ist möglich z. B. Sportfreunde Siegen). Bei Zustimmung aller Gläubiger kann der Club fortbestehen und den Insolvenzplan umsetzen und der Club ist von Altschulden befreit (§§ 244–253 InsO). Der Insolvenzverwalter bleibt bis zum Abschluss des Insolvenzplanes im Amt (§ 268 InsO). Sollte die Gläubigerversammlung den Insolvenzplan nicht akzeptieren, werden alle verbleibenden Vermögensgegenstände veräußert, anschließend an die Gläubiger ausgezahlt und der Club liquidiert (§ 231 InsO).

Neben den sich ergebenden Konsequenzen aus dem Insolvenzrecht sehen die Verbände bestimmte sportliche Sanktionen für insolvente Fußballclubs vor, sofern diese weiterhin am DFB-Spielsystem teilnehmen möchten. Derartige Vorgaben sind nahezu verpflichtend für einen insolventen Club, da außerhalb des DFB-Systems keine alternative Wettbewerbsstruktur existiert, an welcher die Clubs partizipieren und das Geschäftsmodell aufrechterhalten könnten. Bis 2007 war der Zwangsabstieg in den Spielordnungen der DFL, des DFB als auch der Regional- und Landesfußballverbände (im Falle der Eröffnung eines Insolvenzverfahrens oder bei Abweisung mangels Masse) als sportliche Sanktion festgeschrieben (§ 6 Nr. 1 SpO DFB). Im Jahr 2007 änderte zunächst die DFL (1. und 2. Bundesliga) die sportlichen Sanktionen eines Insolvenzverfahrens auf einen Punktabzug von 9 Punkten in der aktuellen Spielzeit ab. Infolge eines Einspruches des VFC Plauen gegen einen Zwangsabstieg (basierend auf einem Konflikt mit der Insolvenzordnung), stellten sowohl der DFB (3. Liga) als auch die Regionalverbände Nord, West, Süd-West, Bayern und Nord-Ost (Regionalligen) zum 01.07.2015 auf die „9-Punkte-Strafe“ um. Hauptargument des Insolvenzverwalters Siemon war, dass der Zwangsabstieg gleichzusetzen sei mit einer Stilllegung als „gravierendes Sanierungshindernis“ und damit rechtlich gegen die Insolvenzordnung verstößt (Siemon, 2015). Es kam zu einer einstweiligen Verfügung und anschließend zu einem Vergleich (LG Berlin 26.02.2015 – 35 O 56/15). Um dieser Diskussion keinen weiteren rechtlichen Raum zu geben, entschieden sich der DFB und die Regionalverbände gegen einen Zwangsabstieg. Seitdem werden Clubs nach Antragstellung auf Eröffnung eines Insolvenzverfahrens 9 Punkte zum Saisonende abgezogen, sofern der Antrag vom Club gestellt wird (§ 6 Nr. 6 SpO DFB). Wird der Antrag jedoch von Seiten eines Gläubigers eingereicht, sind sportliche Konsequenzen erst mit der Eröffnung eines Insolvenzerfahrens durchsetzbar (§ 6 Nr. 1 SpO DFB). Während der Westdeutsche Fußballverband und der Berliner Fußballverband ebenfalls einen Abzug von 9 Punkten festschreiben, gelten Clubs in allen anderen Landesverbänden nach der Eröffnung des Insolvenzverfahrens als direkter Absteiger aus der aktuellen Liga. Verbandsseitige Insolvenzsanktionen sind somit hinsichtlich des Strafmaßes von der Spielklasse abhängig (Tab. 1). Mit Bezug auf die bestehende Rechtsprechung, ist es demzufolge unverständlich (und aus sportlicher Sicht eine eklatante Wettbewerbsungleichheit zwischen Clubs verschiedener Oberligen), dass Landesverbandsregulierungen weiterhin einen Zwangsabstieg vorsehen.

**Tab. 1:** Verbandsspezifische Insolvenzregelungen nach Ligazugehörigkeit

Liga	Stelle	Konsequenz
1. Bundesliga	§ 11 Abs. 5 LO DFL	9 Punkte Abzug
2. Bundesliga	§ 11 Abs. 5 LO DFL	9 Punkte Abzug
3. Liga	§ 6 Abs. 6 SpO DFB	9 Punkte Abzug
RL West	§ 52 Abs. 9 SpO WDFV	9 Punkte Abzug
RL Nord	§ 6 Abs. 9 SpO NFV	9 Punkte Abzug
RL Nord-Ost	§ 6 Abs. 8 SpO NOFV	9 Punkte Abzug
RL Süd-West	§ 6 SpO RLSW	9 Punkte Abzug
RL Bayern	§ 11 Abs. 6 RLO BFV	9 Punkte Abzug
OL Westfalen	§ 52 Abs. 9 SpO WDFV	9 Punkte Abzug
OL Niederrhein	§ 52 Abs. 9 SpO WDFV	9 Punkte Abzug
OL Mittelrhein	§ 52 Abs. 9 SpO WDFV	9 Punkte Abzug
OL Hamburg	§ 16 Abs. 2 SpO HFV	Zwangsabstieg
OL Bremen	§ 25 Abs. 4 SpO BFV**	Zwangsabstieg
OL SH-HO	§ 19a SpO SHFV	Zwangsabstieg
OL Niedersachsen	§ 34 SpO NFV	Zwangsabstieg^a^
OL Bayern Nord	§ 67 Abs. 1 SpO BFV	Zwangsabstieg
OL Bayern Süd	§ 67 Abs. 1 SpO BFV	Zwangsabstieg
OL Nord-Ost Nord	§ 6 Abs. 3 SpO NOFV	Zwangsabstieg
OL Nord-Ost Süd	§ 6 Abs. 3 SpO NOFV	Zwangsabstieg
OL RP/Saar	§ 26 Abs. 1 SpO SWFV	Zwangsabstieg
OL BaWü	§ 6 Abs. 1 SpO WFV	Zwangsabstieg
OL Hessen	§ 16b SpO HFV***	Zwangsabstieg
Berlin (ab 6. Liga)	§ 26a SpO BFV****	9 Punkte Abzug

*LO* Lizenzordnungen, *SpO* Spielordnung, *DFL* Deutsche Fußball Liga, *DFB* Deutscher Fußballbund, *WDFV* Westdeutscher Fußballverband, *NFV* Norddeutscher Fußballverband, *NOFV* Nord-Ost-Deutscher Fußballverband, *RLSW* Regionalliga Süd-West, *RLO* Regionalligaordnung, *BFV* Bayrischer Fußballverband, *HFV* Hamburger Fußballverband, BFV** Bremer Fußballverband, *SHFV* Schleswig-Holsteiner Fußballverband, *NFV* Niedersächsischer Fußballverband, *SWFV* Süd-West-Deutscher Fußballverband, HFV*** Hessischer Fußballverband, BFV**** Berliner Fußballverband, die Oberliga Bayern heißt offiziell Bayernliga

^a^Nachfolgevereine unterste Liga

## Kritische Diskussion verbandsseitiger 9-Punkte-Sanktion

Ausgehend von der aktuellen und einheitlichen *Sanktionspraxis* oberhalb der Oberligen steht jedoch die Frage im Raum, inwiefern Gesetzeskonflikte zwischen InsO und Verbandsregelungen existieren, denn ob die aktuellen Punktesanktionen wirklich rechtlich zulässig sind, ist richterlich weiterhin nicht abschließend entschieden. Zunächst ist festzuhalten, dass die InsO das Ziel verfolgt, eine „bestmögliche“ Gläubigerbefriedigung zu ermöglichen (§ 1 InsO), wobei der Unternehmenserhalt gesichert werden sollte (Fortführungsmaxime). Obwohl Entscheidungen zweier Gerichte über sportliche Sanktionierungen von Insolvenzverfahren vorliegen^8^, wird die Wirksamkeit der einschlägigen Sanktionsklauseln als sehr fraglich in der existierenden Literatur diskutiert (Hörnig & Knauth, 2018; Knauth, 2018). Hauptargument in dieser Debatte ist die Gefährdung einer Unternehmensfortführung, welche sich aus dem Konflikt eines Zwangsabstieges mit der Fortführungsmaxime ergibt (Hörnig & Knauth, 2018; Knauth, 2018). Auch wenn die 9‑Punkte-Sanktion einige Vorteile aus Verbands- und Clubperspektive bieten (kein Zwangsabstieg, transparent für alle, keine Möglichkeit von subjektivem Interpretationsspielraum), so besteht dennoch Kritik hinsichtlich der Willkür der Abzugshöhe, da verbandsseitig bis heute keine öffentliche Erklärung bzw. Motivation der 9 Punkte vorliegt (Hörnig & Knauth, 2018).

Zur empirischen Unterfütterung der Kritik hinsichtlich der Willkürlichkeit eines fixen 9‑Punkte-Abzuges, wurden in Eigenrecherche die realen Ligakonstellationen und Tabellenendsituationen der ersten bis 5. Liga seit Einführung der 3. Liga (2008/2009 bis 2017/2018) zusammengestellt. Im Kern steht die Frage, ob die relative Wirkung eines 9‑Punkte-Abzuges von der Ligazugehörigkeit abhängig ist und somit zu Wettbewerbsnachteilen durch eine Ungleichbehandlung zwischen Clubs verschiedener Ligen führen würde. Im Interesse eines fairen Wettbewerbs aus Verbandssicht sollten die sportlichen Konsequenzen einer Insolvenz für alle Clubs gleichartig und unabhängig von der Liga‑/Verbandszugehörigkeit sein.^9^ Zunächst fällt jedoch auf, dass die durchschnittliche Anzahl von Fußballclubs pro Liga variiert. Da der Punktabzug, soweit er regulatorisch vorgesehen ist, unabhängig von der Ligagröße gilt, wirkt die Strafe in Ligen mit einer geringeren Anzahl an teilnehmenden Clubs härter. Während seit Einführung der 3. Liga die Anzahl der Clubs in den ersten drei Ligen konstant blieb, variierten die Ligagrößen der Regional- und Oberligen deutlich (Tab. 2). In den Regionalligen variierten die Ligagrößen von 16 Teams (fünf Regionalligasaisons) bis zu 20 Teams (zwei Regionalligasaisons). In Bezug auf die Oberligen war die Spreizung noch extremer, wobei die Ligagrößen zwischen 14 (eine Oberligasaison) und 20 Clubs (drei Oberligasaisons) variierten. Wie Tab. 2 zu entnehmen ist, schwankt der relative Punktabzug (9 Punkte im Verhältnis zur maximalen Gesamtpunktanzahl) zwischen 7,9 % relativem Punktabzug (20er-Liga) und 11,5 % relativem Punktabzug (14er-Liga). Diese Existenz einer unterschiedlichen relativen Strafe steht im Widerspruch zur Gleichheit aller Clubs auf einem Ligalevel im DFB-System – unabhängig von der Ligazugehörigkeit.

**Tab. 2:** Anzahl von Clubs je Liga und die relative Strafe bei Eröffnung eines Insolvenzverfahrens

	Anzahl Fußballclubs je Liga (2008/2009 bis 2017/2018*)*						
	14	15	16	17	18	19	20
1. Bundesliga	0	0	0	0	10	0	0
2. Bundesliga	0	0	0	0	10	0	0
3. Liga	0	0	0	0	0	0	10
Regionalligen	0	0	5	0	25	7	2
Oberligen	1	2	32	8	44	7	3
Summe Beobachtungen	1	14	89	8	37	2	1
Max. Gesamtpunkte	78	84	90	96	102	108	114
*Relative 9‑Strafe*	*0,115*	*0,107*	*0,100*	*0,094*	*0,088*	*0,083*	*0,079*

Ungeachtet dieser relativen Verzerrungen, könnte es ferner zu einer ungleichen Strafwirkung kommen, je mehr die Spielstärken innerhalb der Liga ungleich sind. Hierbei könnte die unterschiedliche Wirkung der Punktstrafe auf die letztendliche Aufstiegs- und Abstiegsentscheidung ein Ansatzpunkt sein. Tab. 3 skizziert daher – auf Basis tatsächlicher Punkteverteilungen zum Saisonabschluss in den Spielzeiten 2008/2009 bis 2017/2018 –, welche Konsequenz ein Abzug von 9 Punkten auf das Ligaergebnis jedes einzelnen Clubs von der 2. Bundesliga^10^ bis zur Oberliga gehabt hätte. Tab. 3 differenziert daher zwischen dem tatsächlichen Saisonresultat (Aufstieg, Klassenerhalt, Abstieg) und dem Resultat unter Abzug eines 9‑Punkte-Abzuges. Die dritte Spalte berichtet die prozentuale Abweichung zwischen diesen beiden Szenarien (Differenz in Prozent). Im Fall der 3. Liga gab es somit von 2007 bis 2018 170 Clubs mit einem tatsächlichen Klassenerhalt, von denen jedoch 56 bei einem 9‑Punkte-Abzug abgestiegen wären (56/170 = 32,94 %). Die Wahrscheinlichkeit eines Abstiegs bei Insolvenz trotz sportlichen Klassenerhalts liegt demnach historisch bei 32,94 % in der 3. Liga. Für 20 der 30 *realen* Aufstiegskandidaten hätte ein Insolvenzverfahren mit einem 9‑Punkte-Abzug den Aufstieg verhindert (20/30 = 66,67 %). Bei Betrachtung der Ergebnisse wird daher deutlich, dass ein fixer Abzug von 9 Punkten zu unterschiedlich *harten* Sanktionswirkungen in unterschiedlichen Liganiveaus geführt hätte. Ein 9‑Punkte-Abzug hätte in den Regionalligen die negativsten Konsequenzen im Kampf um den Aufstieg (−76 %) gehabt. Hingegen wäre in der 2. Bundesliga ein starrer 9‑Punkte-Abzug am gravierendsten für Clubs im Abstiegskampf (+39 %) gewesen. In Bezug auf die Konsequenzen der konkreten Insolvenzverfahren seit der Umstellung 2015 haben bis Juli 2020 sieben Clubs die Liga trotz Punktabzugs gehalten, drei Clubs den auch ohne Punktabzug fälligen sportlichen Abstieg realisiert und zwei Teams wären aufgrund des 9‑Punkte-Abzuges sportlich abgestiegen.^11^ Nichtsdestotrotz sollte aus dieser geringen historischen Relevanz (nur zwei Fälle) nicht auf eine geringe zukünftige Irrelevanz geschlossenen werden (Taleb, 2010), da die Zeitreihe zum einen sehr kurz ist und zum anderen der zukünftige Eintritt eines relevanten Falls für den betroffenen Verein von existenzbedrohender Wirkung wäre. Außerdem zeigen die in Tab. 3 berechneten Differenzen die theoretische sportliche Relevanz der 9‑Punkte-Sanktion, welche als zukünftige Relevanzwahrscheinlichkeit der 9‑Punkte-Abzugsregel interpretiert werden kann. Auch sollten theoretische Ungleichheiten ebenso präventiv ausgeschlossen werden wie bereits beobachtete Ungleichheiten, um Rechtsverfahren und Frust im Eintrittsfall vorzubeugen. Weiterhin ist zu erwähnen, dass die nicht abgestiegenen Clubs nicht unbedingt Abstiegskandidaten waren. Alemannia Aachen und VFR Aalen beendeten die Saison 2016/2017 (Regionalliga und 3. Liga) auf dem 7. Platz bzw. 11. Platz und hätten ohne die 9‑Punkte-Sanktion auf dem 4. Platz bzw. 5. Platz abgeschlossen.

**Tab. 3:** Wirkung eines potenziellen 9‑Punkte-Abzuges auf die Ligaendplatzierungen aus Sicht eines jeden Vereins (2008/2009 bis 2017/2018)

	*Eigentlich Aufsteiger*	*Kein Aufsteiger aufgrund 9‑Punkte-Abzug*	*Differenz (%)*
2. Bundesliga	30	20	−66,67
3. Liga	30	20	−66,67
Regionalligen	45	34	−75,56
Oberligen	146	98	−67,12
	*Eigentlich kein Absteiger*	*Absteiger aufgrund 9‑Punkte-Abzug*	*Differenz (%)*
2. Bundesliga	150	59	+39,33
3. Liga	170	56	+32,94
Regionalligen	587	119	+20,27
Oberligen	1358	401	+29,53

Zusammengefasst lässt sich also festhalten, dass der fixe Abzug von 9 Punkten aus empirisch-statistischer Sicht wettbewerbsverzerrend erscheint.

## Fehlanreize und Dysfunktionalitäten

Grundlegend ist ein Insolvenzplanverfahren durch Sonderrestrukturierungsmöglichkeiten (z. B. Schuldenschnitt, Mitarbeiter- und Vertragskündigungen, Gesellschafterumstrukturierungen) eine Chance, ein nachhaltigeres und effizienteres Management in einer Organisation zu etablieren (Maksimovic & Phillips, 1998; Siemon, 2015). Zur Sicherung der Qualität und der Fairness des sportlichen Wettbewerbs sollte ein erfolgreiches Insolvenzplanverfahren daher eigentlich im Interesse des Verbandes und der konkurrierenden Clubs sein. Aus Verbandssicht wird außerdem argumentiert, dass fußballspezifische Insolvenzsanktionen eine Abschreckungsfunktion zur Minimierung risikobehafteten Managements erfüllen und somit risikoaffines/wettbewerbsverzerrendes Verhalten (z. B. Anwerben von Spielern mit eigentlich nur im „Best-case“-Szenario finanzierbaren Gehaltsversprechen) reduzieren.^12^ Zudem erfüllen die sportlichen Sanktionen eine gewisse Filter- und Selektionsfunktion von Clubs in die maximal finanzierbare Liga und könnten – zumindest langfristig – in gewissem Maße einen sportlich ausgeglichenen Wettbewerb fördern (Hoehn und Szymanski, 1999; Szymanski, 2003). Diesbezüglich sollten verbandsseitige Sanktionen durchaus Anreize zu finanziell vorsichtigerem Verhalten setzen – zumindest bei risikoaversen Clubs (z. B. Ausgaben nur in Höhe der Einnahmen, Saisonziel, Klassenerhalt statt Aufstieg). Neben den aufgeführten *Pro-Argumenten* für Sanktionsmechanismen lassen sich – aufgrund der Besonderheiten des Fußballmarktes – jedoch einige Anreize für dysfunktionale Handlungsoptionen identifizieren, welche in der Praxis bereits *Beliebtheit* gefunden haben oder in Zukunft verstärkt genutzt werden könnten.

In diesem Kontext ist zunächst die Möglichkeit einer relativ risikoarmen Entschuldung (stabile Nachfrage nach Fußball, Lock-in-Effekte, lokale Systemrelevanz) über ein Insolvenzverfahren zu diskutieren. Konkret kann die Freisetzung von Ressourcen durch ein Insolvenzverfahren (z. B. geringere Fremdkapitalzinsen, geringere Personalausgaben) im Kampf um sportliches Humankapital einen Vorteil gegenüber Konkurrenzvereinen darstellen. Dieser Umstand kann – entgegen den eigentlichen Verbandsintentionen – die Anreize für ein verantwortungsvolles und nachhaltiges Management senken und zu weiteren Wettbewerbsverzerrungen führen (Dilger, 2009).

Des Weiteren kommunizieren Clubverantwortliche gerne, dass der Club/Vorstand „keine Insolvenz will“.^13^ 2020 leitete der Karlsruher SC sogar ein Mitgliedervotum bezüglich der Antragsstellung eines Insolvenzverfahrens ein. Diese Aussagen haben den Anschein einer *Wahloption*. Derartige Wahloptionen räumt die InsO jedoch nicht ein: Liegen Insolvenzgründe vor, ist nach § 15a InsO – spätestens nach drei Wochen – ein Eröffnungsantrag zu stellen. Tatsächlich ist gerade die Mehrheit der Fußballclubs der dritten bis fünften deutschen Ligen bilanziell (negatives Eigenkapital) überschuldet,^14^ wonach ein Antrag auf Eröffnung eines Insolvenzverfahrens gestellt werden müsste. Dieser Schritt kann jedoch hinausgezögert werden, sofern eine positive Fortführungsprognose vorliegt (§ 19 Abs. 2 InsO). Betroffene Fußballclubs legen jedoch oft den Umstand der unkalkulierbaren sportlichen Zukunft zu einer positiven Fortführungsprognose^15^ nach § 19 Abs. 2 InsO aus, wonach der hypothetische Fall einer positiven sportlichen Entwicklung (z. B. Aufstieg, Spielerverkauf, neuer Sponsor, neuer Investor) die Einnahmen erhöht und die Liquiditätslücke im Planungszeitraum – theoretisch – schließt.^16^ Folglich könnten Fußballclubs immer dann einen Antrag auf Eröffnung eines Insolvenzverfahrens stellen, wenn Überschuldung vorliegt und die Fortführungsprognose negativ *gerechnet* wird. Diese mittelbare *Option* eröffnet die Möglichkeit, einen Antrag auf Eröffnung eines Insolvenzverfahrens dann einzureichen, wenn der Abzug von 9 Punkten ohne relevante sportliche Konsequenzen kompensiert werden kann. Unter Beachtung des Ziels eines fairen Wettbewerbs erscheint eine derartige strategische *Ausnutzung* der Insolvenzrichtlinien kritisch, wenn Clubs ohne entsprechende Puffer diese strategische Option nicht nutzen können. Ferner gelten diese Fehlanreize nicht nur im Abstiegskampf. Auch Aufsteiger könnten sich bei entsprechendem Vorsprung noch vor der neuen Saison sanieren, um dann auf dem Markt für Talente mit mehr Freiheitsgraden agieren zu können.

Ein weiterer Umstand, welcher Fehlanreize zur Ausnutzung der Sanierungsmöglichkeiten über ein Insolvenzverfahren bietet, ist die Tatsache, dass die Anzahl an abgeschlossenen Insolvenzverfahren nicht limitiert ist. Clubs mit langer Tradition und/oder treuen Fans und Stakeholdern, könnten daher ein Insolvenzverfahren vermehrt zur Entschuldung nutzen, während kleinere und neuere Vereine diesen Vorteil nicht in diesem Ausmaß abrufen können. In diesem Sinne ist es daher auch nicht verwunderlich, dass in den letzten 25 Jahren gerade *Kult-Clubs* bereits mehrfach (z. B. KFC Uerdingen, Fortuna Köln, SSV Ulm 1846, Hessen Kassel, Kickers Offenbach, Alemannia Aachen, FC Rot-Weiß Erfurt) Anträge auf Eröffnung eines Insolvenzverfahrens eingereicht haben und weiterhin im Bereich der ersten fünf Ligen aktiv sind.

**Abb. 1:**
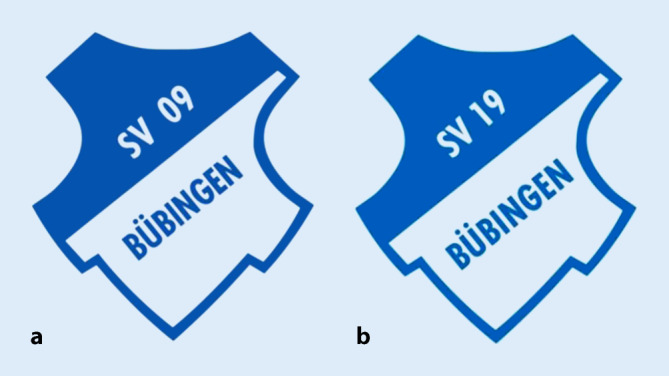
SV 19 Bübingen als nahezu identischer Nachfolgeclub des insolventen SV 09 Bübingen. **a** Liquidierter Club, **b** Nachfolger-Club

Eine weitere dysfunktionale Option ist die Übertragung des Ligastartrechts auf eine Nachfolgeorganisation (Verein oder Kapitalgesellschaft) im Liquidationsfall.^17^ Infolgedessen kann der Spielbetrieb durch den Nachfolgeclub – ohne größere sportliche Konsequenzen – fortgeführt werden, während die insolvente Organisation liquidiert wird. Da die einzige Anforderung an einen Auffangclub ein neuer originärer Club-Name ist (noch nicht im Vereins- oder Firmenregister eingetragen), kann ein Club nahezu ohne sichtbare Veränderung fortbestehen. Ligastartrechte, Personalverträge, Spielerrechte und auch der Clubstandort (Stadion) können übernommen werden. Letzteres fällt immer an die Kommune (oder war nur gepachtet) und wird aufgrund fehlender Mieteralternativen mit hoher Wahrscheinlichkeit wieder an den Nachfolgeclub verpachtet. Auch die für die Insolvenz *verantwortlichen* Funktionäre können ohne konkrete Regulierung die Ämter im Nachfolgeclub besetzen. Im Ergebnis können Fußballclubs mit dieser Maßnahme bestimmte vertragliche Verpflichtungen umgehen. Wie identisch ein derartiger Nachfolgeclub aufgezogen werden kann, zeigt Abb. 1 mit dem Insolvenzbeispiel des SV 09 Bübingen aus dem Saarland. In ähnlich perfider Weise übertrug die insolvente Alemannia Aachen GmbH im Juni 2018 die Spielrechte für 24 h auf den TSV Alemannia Aachen e. V., um diese anschließend an die neue TSV Alemannia Aachen GmbH weiterzugegeben.^18^

Ungeklärt scheint ferner bisher die Frage, inwieweit Fußballunternehmen zunächst Spielervermögen aktivieren müssten, bevor über ein Insolvenzverfahren entschuldet werden kann. Dieser Sachverhalt ist dahingehend kritisch, dass Clubs mit vertraglich gebundenen Nachwuchsspielern oder einem hohen Anteil an ablösefrei erworbenen Spielern bilanziell überschuldet sein können, obwohl Spielerveräußerungen den Insolvenzgrund obsolet machen könnten.^19^ Auch mit qualitativ niedrigerem Spielermaterial ließe sich der Fußballclub durchaus weiterführen und das Ziel eines Überlebens erreichen.^20^ Daher könnten die Spielerrechte der wertvollsten Spieler womöglich nicht (zwingend) betriebsnotwendiges Vermögen sein. Besonders unter Berücksichtigung der besten Gläubigerbefriedigung entsteht die Frage, ob potenziell wertvolle und vertraglich gebundene Spieler nicht veräußert werden müssten bzw. Ablöseangebote unter dem üblichen Marktpreis zu akzeptieren wären. Da bisher keine Regulierungen hinsichtlich eines Veräußerungsgebotes existieren, besteht ein gewisser Fehlanreiz für Clubs mit einem hohen Anteil an stillen Spielerrechtereserven, ein Insolvenzverfahren gezielt zur Entschuldung zu nutzen, obwohl eigentlich noch ausreichend Ressourcen zur Abwendung einer Illiquidität oder Überschuldung vorliegen.

Aufgrund der langen Liste von Clubs mit vorinsolvenzlichen Vereinbarungen in der Vergangenheit^21^ erscheint ferner die fehlende Regulierung eines vorinsolvenzlichen Verfahrens diskussionswürdig. Bisher verhandeln Fußballclubs oft bilateral mit einzelnen Gläubigern über Schuldenstundungen und -verzichte, ohne jedoch ein ganzheitliches Sanierungskonzept (unter Einbindung aller Gläubiger) zur Vermeidung einer Insolvenz zu gestalten. Dieses gibt den Clubs durchaus den Anreiz, größere Restrukturierungen aufzuschieben und weiter risikoaffin (z. B. Transferausgaben, Spielergehälter) zu agieren. Aus Verbandssicht ist somit ein unreguliertes vorinsolvenzliches Verfahren eine potenzielle Quelle von unerwünschten Wettbewerbsverzerrungen, da die Kosten des Spielerkaders eigentlich nicht finanzierbar sind, durch Gläubigerverzichte ohne Restrukturierung jedoch (zumindest mittelfristig) möglich werden.

## Reformierungsmöglichkeiten und Herausforderungen

Ausgehend von den Besonderheiten des Fußballmarktes, bieten sowohl aktuelle verbandsseitige Insolvenzregelungen als auch die Insolvenzordnung Anreize zu einer, aus Verbands- und Wettbewerbersicht, dysfunktionalen Ausnutzung der Rechtsräume. Zur Minimierung von Wettbewerbsverzerrungen bzw. zur Maximierung der Wettbewerbsfairness sind einige Adjustierungen^22^ der Insolvenzparagraphen in den Spielordnungen durch die Verbände denkbar.

Basierend auf der rechtlich kritischen Anordnung von Zwangsabstiegen im Insolvenzfall, sollten alle Landesverbände von Zwangsabstiegsparagraphen absehen und auf den Punktabzugs-Konsens des DFB und der Regionalverbände umstellen. Feste Punktabzugs-Sanktionen führen jedoch zu Benachteiligungen von Clubs verschiedener Ligen. Prozentuale Punktsanktionen wären ein deutlich flexibleres und an die vorherrschende Wettbewerbssituation angepassteres Maß. Die Frage nach einem adäquaten prozentualen Maß erscheint komplex; Tab. 4 zeigt eine Möglichkeit auf. Abgetragen sind die Perzentile^23^ als Anteil an der Gesamtstichprobe der Clubs von 2008 bis 2018, welche bei einer Punktesanktion größer als der Punktepuffer (Spalte 2 und Spalte 4) nicht aufgestiegen bzw. nicht abgestiegen wären. Anschließend sind diese absoluten Punkte ins Verhältnis zur Gesamtpunktzahl in der beobachteten Saison gesetzt (Spalte 3 und Spalte 5). So hatten bspw. 35 % (Perzentil = 0,35, Spalte 1) der sportlichen Aufsteiger 5 oder weniger Punkte vor dem Nichtabstieg (65 % mehr als 5 Punkte). Eine Sanktion von 6 Punkten (>5,6 % relative Punkte, Spalte 3) hätte also bei 35 % der eigentlichen Aufsteiger den Aufstieg im Insolvenzfall verhindert. In Bezug auf die Absteiger hatten 35 % (Perzentil = 0,35, Spalte 1) der Nicht-Absteiger 11 Punkte Abstand auf den ersten sportlichen Absteiger (65 % mehr als 11 Punkte). Eine Sanktion von 12 Punkten (>10,8 %, Spalte 5) hätte also 35 % der Nicht-Absteiger in den Abstieg gezwungen. Die letztendlich gewählte relative Punktabzugsgrenze würde von verbandseigenem Anspruch abhängen. Dazu müssten die Verbände klären, wie viele Absteiger (oder Nicht-Aufsteiger) im Sinn des Verbandsanspruchs tolerierbar wären, um die sportlichen Gleichgewichte und den Wettbewerb nicht unnötig stark zu strapazieren. Sollten z. B. maximal 15 % (Spalte 1) der Clubs bei einer Insolvenzsanktionierung „hart“ (Abstieg trotz sportlichen Klassenerhaltes) bestraft werden, so dürfte die relative Strafe nicht größer als 5,9 % (Spalte 5) der Maximalpunktzahl der Liga sein. Dabei sollte natürlich nicht unberücksichtigt bleiben, dass der Abzug von 9 Punkten einen Einfluss auf das (strategische) Verhalten und die Performance der anderen Clubs haben könnte und sich die relativen Strafen dementsprechend auch vom Spieltag der Insolvenzeröffnung abhängig sein könnten (Elaad, Krumer, & Kantor, 2018).

**Tab. 4:** Anteil an Clubs und Auswirkung von Punktabzügen (2. Bundesliga bis Oberligen; 2008/2009 bis 2017/2018)

Perzentil	Punktepuffer absolut vor Nicht-Aufstieg	Punktepuffer relativ^a^ vor Nicht-Aufstieg	Punktepuffer absolut vor Abstieg	Punktepuffer relativ^a^ vor Abstieg
0,01	1	0,010	1	0,010
0,05	2	0,020	3	0,029
0,10	2	0,022	4	0,044
0,15	3	0,029	6	0,059
0,20	4	0,039	7	0,069
0,25	4	0,044	8	0,083
0,30	5	0,049	10	0,098
0,35	5	0,056	11	0,108
0,40	7	0,069	14	0,135
0,45	7	0,069	14	0,135
0,50	8	0,078	15	0,147
0,55	8	0,083	17	0,167
0,60	9	0,088	18	0,176
0,65	10	0,098	20	0,196
0,70	11	0,108	21	0,216

^a^Verhältnis von absolutem Punktepuffer zur maximalen Punktgesamtzahl der jeweiligen Liga und Saison

Möglich ist ferner, einem sportlichen Aufsteiger bei Eigenantrag auf Eröffnung eines Insolvenzverfahrens vor dem letzten Spieltag einen Punktabzug zur nächsten Saison aufzuerlegen (bzw. Wahlrecht auf Seite des Verbandes), um eine risikolose (ausreichende Punkte) *Schnellsanierung* vor dem Aufstieg auszuschließen.

Als nächste Voraussetzung für die Fortführung eines insolventen Clubs im Ligasystem könnte zunächst die Auflage gemacht werden, aktuelle vertraglich gebundene Spieler auf dem Markt anzubieten (und bei Verkauf durch ablösefreie Spieler zu ersetzen), um wirklich alle Ressourcen freizumachen und somit eine *bilanzielle Aktivierung* versteckter Spielerrechte zu erwirken. Es kann natürlich zu keinem Zwangswechsel kommen.^24^ Vorstellbar wäre jedoch die Notwendigkeit, die Spielerrechte auf dem Markt anzubieten und auch Ablöseangebote am unteren Ende des Marktwertes zu akzeptieren (z. B. Auktion), sofern der Spieler dem Wechsel zustimmt. Dem würde es auch nicht widersprechen, dass ein faktischer Übergang des Spielerrechts erst in der nächsten Wechselperiode erfolgen kann, da eine Vertragsunterzeichnung außerhalb des Transferfensters auch zu Forderungen gegenüber dem aufnehmenden Club führen könnte. Fehlende Nachweise über einen Veräußerungsversuch könnten mit zusätzlichen Punkt‑, Transfer‑, Nichtaufstiegs- oder Spielrechtstrafen sanktioniert werden.^25^

Relativ einfach könnte die Übertragung von Ligastartrechten auf Nachfolgeclubs unterbunden werden. Wie am Beispiel der Spielordnung des Fußballlandesverbandes Niedersachsen, ist eine derartige Regulierung durchaus möglich und wird in der Praxis eingesetzt.^26^ Hier könnten DFL, DFB, Regionalverbände und weitere Landesverbände nachziehen und ein generelles Übertragungsrecht von Ligaspielrechten (oder im Spezialfall Insolvenz) untersagen.

Eine aus ökonomisch-rechtlicher Sicht durchaus spannende Herausforderung ist die am 16.07.2019 in Kraft getretene EU-Regulierung zu vorinsolvenzlichen Sanierungsverfahren (Richtlinie 2019/1023 über Restrukturierung und Insolvenz). Diese Richtlinie (Umsetzungszeitraum 2 Jahre) gibt Mitgliedstaaten vor, ein vorinsolvenzliches Verfahren in die nationalen Rechtsordnungen zu implementieren, mit dessen Hilfe drohend zahlungsunfähige Schuldner eine präventive finanzielle Restrukturierungsmöglichkeit erhalten. Kernelement ist ein Sanierungsplan (vergleichbar mit Insolvenzplan), welcher mit Mehrheit der Gläubiger beschlossen wird und i. d. R. umfassende Forderungsstundungen und -erlasse vorsieht. Nach Implementierung in nationales Recht werden sich auch die Fußballverbände mit der Frage auseinandersetzen, inwiefern vorinsolvenzliche Sanierungsverfahren sportlich sanktioniert werden sollten. Es wäre wohl ein konsequenter Schritt. Ob es ein richtiger wäre, steht auf einem anderen Blatt, da die EU-Richtlinie nur das Ziel einer Insolvenzvermeidung (durch Sicherung von Ressourcen und Werten) verfolgt (Richtlinie (EU) 2019/1023 Nr. 1).

## Fazit

Neben der Insolvenzordnung regeln in Deutschland gesonderte Insolvenzparagraphen in den Spielordnungen der Fußballverbände die sportlichen Konsequenzen eines Insolvenzverfahrens von Fußballclubs, wobei Zwangsabstieg (einige Landesverbände) und ein fester 9‑Punkte-Abzug (DFB, Regionalverbände und einige Landesverbände) aktuell Anwendung finden. Jedoch widerspricht der in einigen Landesverbänden festgeschriebene Zwangsabstieg im Insolvenzfall sowohl der aktuellen Rechtsprechung als auch dem Punktabzugs-Konsens von DFB und Regionalverbänden. Ein fester Abzug von 9‑Punkten führt jedoch zu einer Ungleichbehandlung zwischen Clubs verschiedener Ligen, weshalb ligaangepasste Punktabzugsgrenzen definiert werden sollten. Generell öffnet der Fortbestand im deutschen Ligasystem während und nach einem Insolvenzverfahren verschiedene Anreize zu – aus Verbandssicht – dysfunktionalem Verhalten der Clubs, welche durch die Besonderheiten des Sportmarktes gegeben sind. Die Verbände sollten daher die bisherigen Insolvenzparagraphen überdenken (gerade mit Blick auf die kommende Neuregulierung eines vorinsolvenzlichen Verfahrens nach EU-Richtlinien und der größeren Reformoffenheit durch die COVID-19-Pandemie) und auf allen Ebenen der deutschen Fußballverbandspyramide vereinheitlichen.

## Footnotes

1 Während in der 1. Bundesliga seit 1995 keine Anträge auf Eröffnung eines Insolvenzverfahrens eingereicht wurden, waren es in der 2. Bundesliga nur zwei. Entsprechend der Modellschätzungen von Szymanski und Weimar (2019) lag die Insolvenzwahrscheinlichkeit damit in der 3. Liga 4,1 % Prozentpunkte (28 Fälle) und in der 4. Liga 1,4 % Prozentpunkte (57 Fälle) höher als in der 2. Bundesliga (1995–2018). Die geringere Insolvenzwahrscheinlichkeit in der 4. Liga – trotz einer höheren Anzahl an Fällen – basiert auf dem größeren Team-Pool in den 4. Ligen.

2 Zudem erscheint der Anteil von zurückgenommenen Anträgen auf Eröffnung eines Insolvenzverfahrens im Fußball deutlich höher (17 % statt 4 %) als in klassischen Wirtschaftsbranchen (Szymanski & Weimar, 2019).

3 Bereits 1971 schrieb Sloane (1971, p. 122): „majority of league clubs operate at a loss and only remain solvent through income derived from non-footballing activities.“

4 Dies wäre ungefähr so, als würde eine internationale Fluglinie Insolvenz anmelden, dürfte anschließend nur noch auf dem europäischen Kontinent anbieten, aber die Kunden wären verpflichtet, zum Hinflug mit einer Airline immer den Rückflug mit der insolventen Fluglinie zu buchen – die Nachfrage wäre also sicher.

5 Konkret bedeutet dies im Vergleich zu Nicht-Sportmärkten, dass die Nachfrage von einem zum anderen Tag wegbrechen kann (Abstieg), jedoch wieder in der nahen Zukunft spontan auf das alte Niveau steigen kann (Aufstieg). Das Argument, dass der ganze Fußballmarkt wegbrechen könnte, erscheint, wenn auch theoretisch möglich, von marginaler Wahrscheinlichkeit. Selbst während der COVID-19-Pandemie war die Nachfrage nach Fußball vorhanden, konnte jedoch durch die gesetzlichen Maßnahmen nicht bedient werden. Die Nachfrage ist dann jedoch teilweise auf Ligen im Ausland übergesprungen (Weimar, 2020). Kay Dammholz (Experte Sportrechtevermarktung, Geschäftsführer der Agentur SASS Media) äußerte sich am 24.06.2020 im „Sports-Maniac Podcast“ passend: „Fußball ist eigentlich der einzige Sport, der Krise richtig trotzen kann. Das hat er jetzt auch bewiesen, da er kaum Federn lässt, trotz Corona.“

6 Eine weitere – jedoch in der nachfolgenden Diskussion nur mittelbar relevante – Besonderheit ist die „50+1“ Regel, wonach der Basisverein immer 50 % + 1 Stimme Beteiligung an einer ausgegliederten Kapitelgesellschaft haben muss (Bauers & Hovemann, 2019).

7 In der Vergangenheit haben 17 % der Clubs nach Einreichung eines Antrages auf Eröffnung eines Insolvenzverfahrens noch ausreichend Liquidität akquirieren können und somit die Eröffnung des Insolvenzverfahrens verhindert (Szymanski und Weimar, 2019). Möglich war dies sowohl durch kurzfristige *Retteraktionen* als auch durch Stundung von Gläubigeransprüchen. Gerade durch neuere onlinebasierte Möglichkeiten fallen die Transaktionskosten für kurzfristiges Crowdfunding und -lending (inkl. Fan-Anleihe) stetig (Huth, 2018; Weimar & Fox, 2012).

8 Vgl. OGH Wien (Beschl. v. 27.11.2017) für Zwangsabstiegsklausel in Österreich und das LG Berlin (26.02.2015 – 35 O 56/15) für eine 9‑Punkte-Sanktionierung in Deutschland. Das LG Berlin verhandelte dabei eigentlich über die Zulässigkeit des Zwangsabstiegs, musste jedoch aufgrund eines Vergleichs keine Entscheidung aussprechen. Allerdings wird hinsichtlich der Verbandsregelungen ausgeführt: „wobei in Rechtsprechung und Literatur umstritten ist, ob diese Regelung rechtmäßig ist“ (LG Berlin 26.02.2015 – 35 O 56/15).

9 Sicherlich lässt sich in gewissem Umfang argumentieren, dass ein föderales System mit unterschiedlichen Regulierungen auf Regional- und Landesverbandsebene auch Vorteile durch regional angepasste Regelwerke bietet. Aufgrund der Aufstiegsregelungen in bundesweiten Ligen, fordert der DFB jedoch grundsätzlich gleiche Ausgangsvoraussetzungen und Regeln für die Clubs in den unteren Ligen (Wagner, Preuss, & Schu, 2019), weshalb gerade Insolvenzregulierungen nicht unterschiedlich wirken sollten. An dieser Stelle sei jedoch auch daraufhin gewiesen, dass Teams in kleineren Ligen auch bei den zu erwerbenden Punkten benachteiligt.

10 Zur Komplexitätsreduktion wurde die 1. Bundesliga aufgrund unterschiedlicher Startplätze für Qualifikationen zu verschiedenen Europapokalwettbewerben ausgeschlossen. Zudem lag bisher kein Insolvenzfall seit 1995 in der 1. Bundesliga vor.

11 Ohne sportliche Konsequenz Klassenerhalt: 1. FC Kaiserslautern, Wacker Nordhausen, Westfalia Herne, Viktoria Berlin, Alemannia Aachen, VFR Aalen, Kickers Offenbach; ohne sportliche Konsequenz Abstieg: Chemnitzer FC, Rot-Weiß Erfurt, FSV Frankfurt; Konsequenz Abstieg statt Klassenerhalt: Hessen Kassel, Wuppertaler SV (lediglich ausgesetzt wegen COVID-19-Nicht-Abstiegs-Regelungen); Konsequenz Klassenerhalt statt Aufstieg: –).

12 Dies gilt auch, obwohl die Verbände selbst von einer Insolvenz eines ligaangehörigen Vereins nur unmittelbar betroffen sind; es sind vielmehr die Konkurrenzvereine, welche durch das Merkmal der assoziativen Konkurrenz die Folgen (qualitativ sinkender Wettbewerb) einer Insolvenz tragen müssen.

13 Beispiele: Markus Fauser (12.06.2017, Geschäftsführer 1860 München): „Wir wollen keine Insolvenz, wir wollen den Verein sanieren.“; Vereinssprecher Wacker Nordhausen (03.12.2019): „Eine Insolvenz wolle man aber noch nicht anmelden, man arbeite derzeit an einer anderen Lösung“; Josef Schnusenberg (29.07.2019, Aufsichtsrat SG Wattenscheid 09): „Nach Durchsicht der Unterlagen hat der Aufsichtsrat entschieden, dass es bei der SG 09 Wattenscheid keine Insolvenz geben wird.“

14 Vgl. https://www.kicker.de/734605/artikel.

15 Eine positive Fortführungsprognose ist gegeben, wenn auf mittlere Sicht (in der Regel das laufende und das folgende Geschäftsjahr) die Überwindung der Überschuldungssituation überwiegend wahrscheinlich ist.

16 Beispiele für eine derartige Argumentation einer positiven Fortführungsprognose finden sich öffentlich in Anleiheprospekten vergangener Fan-Anleihen wie z. B. Hansa Rostock (2011), 1. FC Kaiserslautern (2013), Arminia Bielefeld (2011) oder des MSV Duisburg (2013). Konkret wird dies am Wortlaut des Abschlussberichtes für die Hertha BSC Anleihe 2010, welcher sich in sehr ähnlicher Form in allen Abschlussberichten mit negativem Eigenkapital finden lässt: „Die Emittentin ist bilanziell überschuldet. Nach Auffassung der Emittentin liegt eine materielle Überschuldung nicht vor, da von einer positiven Fortbestehensprognose ausgegangen wird“ (Anleiheprospekt Hertha BSC, 2010, S. 12).

17 Beispiele: 1. FC Eschborn zu SC Eschborn (2015/2016); Torgelower Greif zu FC Greif (2012/2013); SpVgg Weiden zu Spielvereinigung Weiden 2010 (2010/2011); 1. FC Eintracht Bamberg zu Eintracht Bamberg 2010 (2009/2010); Dresdner SC Fußball 98 zurück in den Verein Dresdner SC (2005/2006).

18 In einer gemeinsamen Presseerklärung von Insolvenzverwalter und Vorstand ist zu lesen: „Die Alemannia ist damit von Altlasten befreit und kann sich mit Hochdruck darauf konzentrieren, zum einen ein schlagkräftiges Team für die kommende Regionalliga-Saison zusammenzustellen […]“ – Reue und Überdenken vergangener Prozesse klingen anders. https://www.alemannia-aachen.de/aktuelles/nachrichten/details/Erklae-rung-zum-Stand-des-Insolvenzverfahrens-24662c/.

19 Beispielhaft sei hier auf das Insolvenzverfahren des VFR Aalen aus der Saison 2016/2017 hingewiesen. Der Club stellte am 14.02.2017 einen Antrag auf Eröffnung eines Insolvenzverfahrens. Der SWR schrieb: „Damit wollte der Verein die 3,6 Mio. € Schulden loswerden, die sich aufgehäuft hatten und die Zukunft des VFR gefährdeten.“ (https://www.swr.de/sport/die-chronologie-der-insolvenz-des-vfr-aalen/-/id=1208948/did=19257188/nid=1208948/1hflk6x/index.html) Zum 29.06.2017 war das Insolvenzplanverfahren abgeschlossen und der VFR schuldenfrei. In der Folgesaison 2017/2018 standen noch 15 Spieler im Kader und 2018/2019 noch 9 der ehemaligen 28 Spieler – ein Großteil der Spielerrechte wurde demnach nicht veräußert.

20 Grundsätzlich wird in der Überschuldungsfeststellung der Liquiditätsfall gerechnet und daher stille Reserven aufgelöst. Spielerrechte sind jedoch im Liquiditätsfall immer wertlos (Lambrecht & Reiter, 2013), da es sich zum einen um Arbeitsverträge handelt und zum anderen Spieler bei Liquidierung des alten Clubs immer ablösefrei wechseln können.

21 Beispiele: Carl Zeiss Jena „erließ“ 2018 einem Investor die Schulden mit einem Besserungsschein; Bielefeld verhandelte 2018 eine aus Steuergeldern finanzierte Bürgschaft; Hansa Rostock verhandelte 2018 einen Schuldenerlass mit dem Investor; 1860 München verhandelte 2017 eine Stundung mit einem Investor zur Insolvenzvermeidung. 2019 verhandelte der 1. FC Kaiserslautern mit einem Konsortium aus lokalen Investoren und einem externen Bauinvestor aus Luxemburg über die Rettung des Clubs – für letzteren wurde sich (basierend auf nur einfacher Mehrheit) entschieden. Bereits 2018 gab es Verhandlungen mit der Stadt Kaiserslautern über eine Reduzierung der Stadionmiete für zwei Jahre – von ursprünglich 3,2 Mio. € auf 2,4 Mio. € bei Klassenerhalt und 0,4 Mio. € bei einem Abstieg in die 3. Liga. Aktuell wird um eine weitere Verlängerung verhandelt.

22 Es könnte natürlich auch mit der kompletten Abschaffung von sportlichen Sanktionsklauseln argumentiert werden, denn solange Gläubiger weiterhin Geld injizieren und Restrukturierungen mehrheitlich zustimmen, scheint es für bestimmte Marktteilnehmer vorteilhaft zu sein. Diesem Argument, eines auf Verbandsseite insolvenzrechtlich unregulierten Marktes, ist jedoch die Ausnutzung der lokalen Systemrelevanz entgegenzuhalten.

23 0,25 = unterhalb des Wertes liegen 25 % aller Fälle und 75 % darüber, 0,5 = Median = 50 % der Fälle liegen darunter.

24 Jedoch zeigt die Vergangenheit, dass Spieler bei absehbaren Zahlungsschwierigkeiten die eigene Unsicherheit häufig durch einen vorzeitigen Wechsel reduzieren.

25 Grundsätzlich könnte auch eine Anpassung der InsO, hinsichtlich nicht betriebsnotwendigem und nicht aktiviertem Vermögen, motiviert werden.

26 Vgl. SpO NFV §18 Nr. 5 vom Juni 2019: „Neu in den Verband aufgenommene Vereine werden grundsätzlich mit ihren Mannschaften der untersten Klasse ihres Kreises zugeteilt. Dies gilt auch für Vereine, die sich als Nachfolgeverein eines im Insolvenzverfahren liquidierten Vereines darstellen.“
